# Comparative analyses in transcriptome of human granulosa cells and follicular fluid micro-environment between poor ovarian responders with conventional controlled ovarian or mild ovarian stimulations

**DOI:** 10.1186/s12958-022-00926-1

**Published:** 2022-03-21

**Authors:** Xiaoping Liu, Huisi Mai, Panyu Chen, Zhiqiang Zhang, Taibao Wu, Jianhui Chen, Peng Sun, Chuanchuan Zhou, Xiaoyan Liang, Rui Huang

**Affiliations:** Reproductive Medicine Research Center, the Sixth Affiliated Hospital of Sun Yat-Sen University, Guangzhou, China

**Keywords:** Transcriptome analysis, Follicular fluid micro-environment, Mild ovarian stimulation, Controlled ovarian stimulation, Poor ovarian response, Granulosa cells, TGF-β signaling pathway

## Abstract

**Background:**

Both mild and conventional controlled ovarian stimulation are the frequently used protocols for poor ovarian responders. However, there are some debates about which treatment is better. Moreover, little is known about the follicular physiology after the two ovarian stimulation protocols. This study was intended to investigate the features in granulosa cells and follicular fluid micro-environment after the two different ovarian stimulation protocols in poor responders.

**Methods:**

Granulosa cells RNA were sequenced using Illumina Hiseq technology. Specific differently expressed genes and proteins were verified by real-time quantitative PCR and Western blot analysis. Moreover, hormone and cytokine concentrations in the follicular fluid were measured by electrochemiluminescence immunoassay and enzyme-linked immunoabsorbent assay. The correlation between the results of molecular experiments and the laboratory outcomes were analyzed by Spearman correlation analysis.

**Results:**

The differentially expressed genes between the two groups were involved in 4 signaling pathways related to the follicular development; three proteins pertinent to the TGF-β signaling pathway were expressed differently in granulosa cells between the two, and the constituents in the follicular fluid were also different. Further, a correlation between the TGF-β signaling pathway and the good-quality embryo was observed.

**Conclusions:**

The present study made a comparison for the first time in the transcriptome of human granulosa cells and the follicular fluid micro-environment between poor responders with the conventional controlled ovarian stimulation or the mild ovarian stimulation, showing that the TGF-β signaling pathway may correlate with the good-quality of embryos in the mild group, which may be instrumental to the choice of optimal management for IVF patients.

**Supplementary Information:**

The online version contains supplementary material available at 10.1186/s12958-022-00926-1.

## Background

In in vitro fertilization (IVF) practice, a noticeable proportion of women receiving the conventional controlled ovarian stimulation (COS) undergo poor ovarian response (POR), suffering low numbers of retrieved oocytes and transferrable embryos, and low pregnancy rate [1]. The situation of POR patients is critically challenging the ART clinicians although many adjunctive strategies for the COS protocol have been developed, e.g., increasing gonadotropin (Gn) dose, supplementing with exogenous luteinizing hormone (LH), decreasing gonadotropin-releasing hormone agonist (GnRH-a) dose and using adjunctive growth hormone [2–5]. Recently, the mild ovarian stimulation highlights in clinicians’ focus and is favored by some owing to its lower economic burden on patients. However, it remains debatable which protocol is clinically much better, thus, it is necessary to investigate the different influence on the follicular development under the conditions of the conventional COS and the mild ovarian stimulation. The major difference between the conventional COS and the mild ovarian stimulation lies on the daily used dosage of Gn, a key regulator in the follicular development. In vitro experiments showed that an appropriate dose of Gn stimulated follicle growth while a very high dose of Gn led to a decreased follicle survival and the contemporary accumulation of hazardous metabolic products [6], which even made detrimental effects on oocytes and embryos [7–9]. Yet, the mild ovarian stimulation, aiming at a more physiological response, may attenuate such kind of damage.

A series of architectural transformations in follicles during folliculogenesis depend on a complicated and subtle cross-talking mechanism among the three of granulosa cells, the follicular fluid (FF) micro-environment and oocytes. Some studies reported that granulosa cells affected the growth, development [10], energy uptake [11] and transcription process of the oocyte [12]. Besides, the FF micro-environment, the most immediate niche rich in various hormones and cytokines, acts as important communication media in the antral follicle and transports nutrients [13] for the developing oocytes. Thus, different stimulation protocols, influencing the gene expression in granulosa cells and the FF micro-environment differently [14, 15], may have a significant effect on the cell signaling and on the oocyte quality potentially [16]. Because it is infeasible to consume oocytes freely from patients, the granulosa cell and the FF represent two indispensable factors to fathom folliculogenesis. Theoretically, the transcription information from granulosa cells, which are the somatic cell most adjacent to the oocyte, may mirror the developmental competence of the associated oocyte. Therefore, comparing the FF micro-environment and the gene expression of granulosa cells in POR patients with different ovarian stimulation protocols is of great significance for understanding the effects of different protocols on the follicular physiology.

Here, aiming to provide reliable physiological data for selecting optimal management for POR patients, we compared the pertinent data or measurements of granulosa cells, the FF micro-environment and oocytes from POR patients treated with the conventional COS and the mild ovarian stimulation.

## Methods

### Study design and participants

The prospective study, conducted at the Reproductive Medicine Research Center, the Sixth Affiliated Hospital of Sun Yat-sen University, was approved by the Ethics Committee of Reproductive Medicine and Prenatal Diagnosis, the Sixth Affiliated Hospital of Sun Yat-sen University (2019ZSLYEC-002S), and all participants rendered written informed consent.

The POR patients, recruited according to the Bologna criteria [17], received either the mild ovarian stimulation (the mild group) or the conventional COS (the COS group) for the ART treatment. Patients in the mild group, starting with 5 mg of letrozole (Letrozole Tablets, Hengrui Medicine, China) daily from the 3^rd^ to 7^th^ day of the menstrual cycle in conjunction with the injection of 150 IU of recombinant follicle stimulation hormone (FSH) (Puregon, MSD, Germany) on the 4^th^ and 6^th^ day, were administered 150 IU of recombinant FSH since the 8^th^ day of the menstrual cycle until the day of human chorionic gonadotropin (hCG) administration. And when the serum estradiol (E_2_) level was ≥ 200 pg/ml, a gonadotropin-releasing hormone antagonist (GnRH-ant, Cetrotide, Merck-Serono, Switzerland, or Orgalutran, MSD, Germany) was given subcutaneously (0.25 mg/day). The COS group was instituted in terms of a GnRH-a stop protocol with a 14-day mid-luteal down-regulation (0.05 mg/day; Triptorelin, Ferring, Germany), followed by daily stimulation with 300 IU of recombinant FSH (Puregon, MSD, Germany). The final maturation of oocytes was induced with 0.25 mg of hCG (Ovidrel, Merck Serono S.p.A., Italy) when at least one leading follicle reached 18 mm, or 3 follicles reached 17 mm in diameter. Oocyte retrieval was performed transvaginally under ultrasound guidance 36–37 h past the hCG injection. Standard IVF or ICSI was performed as appropriate. The patients’ demographic and clinical characteristics, including age, body mass index (BMI) and the serum basal sexual hormone levels, were recorded for following applications.

### Embryo evaluation

Embryo evaluation methods are as described previously [18]. Day-3 embryo morphology was scored according to blastomere number, the degree of fragmentation and extent of asymmetry [19]. Briefly, each embryo was assigned a fragmentation score of 0%, 1–9%, 10–25%, 26–50%, or > 50%, and an asymmetry score of perfect, moderate or severe asymmetry. A good-quality embryo was defined as having ≥ 6 cells with < 10% fragmentation and either no asymmetry or moderate asymmetry. A transferrable embryo was defined as having ≥ 4 cells with < 26% fragmentation and either no asymmetry or moderate asymmetry.

The quality of blastocyst stage embryos was assessed according to the criteria of Gardner and Schoolcraft [20] based on the degree of expansion and hatching status of the blastocoel cavity (1-6), the size of the inner cell mass (A–C) and the development of the trophectoderm (A–C). A good-quality blastocyst was defined as a quality score ≥ 3BB.

### Collection of follicular fluid (FF) and granulosa cells

The FF and granulosa cells were obtained via ultrasound-guided transvaginal oocyte retrieval. The FF, collected from the largest follicle, was transferred to a 2-ml cryogenic vial (Corning, New York, USA) and then stored at -80℃ for further analyses of hormones and cytokines. The FF from the remaining follicles, pooled in a 50-ml conical centrifuge tube (Corning, New York, USA) after removal of oocyte-cumulus complexes, was centrifuged for 5 min at 500 g for the granulosa cell isolation. The granulosa cells were washed three times with cold sterile phosphate buffered saline (PBS) at 500 g for 5 min at room temperature (RT). Three ml of PBS were added for resuspending the cells and then 3 ml of Ficoll (Tianjin HaoYang Biological Manufacture Co., Ltd, Tianjin, China) were carefully added along the tube wall for gradient centrifugation. The samples were centrifuged at 1000 g for 25 min at RT for the recollection of granulosa cells, which, appearing in the middle interface, were pipetted and transferred to a 1.5-ml Eppendorf tube sitting on ice. Then, 200 μl of cold sterile PBS and 600 μl of red blood cell lysis buffer (CoWin Biosciences, Beijing, China) were added and mixed by inversion, and placed on ice for 15 min. After centrifugation at 1000 g for another 10 min at RT, the granulosa cells were washed again with cold sterile PBS for final centrifugation, and the cell precipitates were stored at -80℃ until RNA extraction [21–23]. Preceding the study, we performed the identification experiments on the extracted cells (Fig. S1).

### RNA extraction

For independent extraction of the total RNA, the granulosa cells sampled from each individual patient were treated and incubated with 500 μl of TRIzon reagent according to the instruction (CoWin Biosciences, Beijing, China). Then, 100 μl of chloroform were added to the solution and mixed thoroughly. After phase separation and centrifugation, the aqueous layer was carefully pipetted into another RNase-free tube, and 500 μl of isopropanol were added to the aqueous phase and incubated for 10 min at RT for the following centrifugation and precipitation of the total RNA. The precipitate was resuspended in 500 μl of 75% ethanol and centrifuged to get RNA pellet. The RNA purity and integrity were measured by using the Nanodrop ND-1000 (NanoDrop Technologies, Wilmington, USA) and the Agilent 2200 TapeStation (Agilent Technologies, Santa Clara, USA), respectively.

### cDNA Library construction and transcriptome sequencing

Fragmented RNAs, approximately 200 bp in average, were subjected to the first strand and second strand cDNA synthesis followed by adaptor ligation and PCR enrichment with a low-cycle according to instructions of NEBNext® Ultra™ RNA Library Prep Kit for Illumina® (New England Biolabs, Ipswich, USA). The purified library products were evaluated using the Agilent 2200 TapeStation and Qubit3.0 (Life Technologies, Carlsbad, USA). The libraries were paired-end sequenced (PE150, sequencing reads = 150 bp) at Guangzhou RiboBio Co., Ltd (Guangzhou, China) using the Illumina Hiseq X-ten platform.

### Gene expression analysis

The differential expression analysis of the two groups was performed using the DEGSeq2 R package (V1.18.1). DEGSeq analysis was used to provide statistical routines for determining differentially expressed genes (DEGs) using a model based on the negative binomial distribution. *P*-values were adjusted (*P*_*adj*_), which was calculated via the Benjamini–Hochberg method to exclude false-positive results. Genes with *P*_*adj*_ < 0.05 were defined as being differentially expressed. All differentially expressed genes were selected for the GO (Gene Ontology) and the KEGG (Kyoto Encyclopedia of Genes and Genomes) analysis. The GO enrichment analysis was performed using the KOBAS3.0 software. GO terms with corrected *P*-values less than 0.05 were considered to be significantly enriched. Enrichment of DEGs in the KEGG was analyzed using the KOBAS3.0 software.

### Real-time quantitative PCR (RT-qPCR) analysis

The significant DEGs were confirmed by RT-qPCR. The primers (Table 1) were designed and synthesized by Sangon Biotech (Shanghai, China) using sequence information obtained from the NCBI Gene Database (https://www.ncbi.nlm.nih.gov/gene/) and blasted (https://blast.ncbi.nlm.nih.gov/Blast.cgi). The total RNA from granulosa cells was reverse-transcribed into cDNA using PrimeScript™ RT Master Mix (Perfect Real Time) (Takara, Kusatsu, Japan). RT-qPCR was performed on LightCycler® 480 System (Roche, Basel, Switzerland) using 2 × RealStar Green Fast Mixture (GenStar, Beijing, China). All the genes were normalized to the gene of glyceraldehyde-3-phosphate dehydrogenase (GAPDH), and results expressed as relative fold changes using the 2^–ΔΔCT^ method.

**Table 1:** Primers used for RT-PCR

Genes	Forward primer sequence (5’-3’)	Reverse primer sequence (5’-3’)
*GAPDH*	GGAGCGAGATCCCTCCAAAAT	GGCTGTTGTCATACTTCTCATGG
*CREB3L3*	CACCTGTGTCGCAGTCCTGTTG	CGGGAGGCAGCATCGTTGTG
*NPR1*	GCCACCATTCCAGCAGATCCG	CCGCTCCTCCACCAGTTCCTC
*TRPC6*	TCCTTGTGGTCCTTGCTGTTGC	GAAGGAGGCTGCGTGTGCTAC
*ATP1A4*	CCTGGAGGCGGTGGAGACG	GCGACGGTCATGCGGTTCTG
*ADRA1D*	GTCTTCGTGCTCTGCTGGTTCC	GCCGAGCCAGAAGATGACCTTG
*CACNA1D*	TCCATCGCTTCGCTGTTGCTTC	AAGGTGCTCCGCTTGGTTTGC
*DAN*	TCCCCAACACCTTCCCACAGTC	GGCACTCCAGCGTCACAATCTC
*PITX2*	ACACCATCTCCGACACCTCCAG	GTCCGCTGCCGCCTTTGC
*ALK7*	TGTGACCGCCTCTGGATCTGG	GCCACATCTTCCCCACACCATC
*CCL4*	GAAGCTCTGCGTGACTGTCCTG	GGCGGTGGGAGGGTCTGAG
*VEGI*	GGCCGAGGATCTGGGACTGAG	GGAGCAACACCAGGCAGCAG
*IL21R*	GTGGCTTTGTGGGCTCTGACTG	CGAAAGTGGCGGAGGAATGACC
*TGFβ2*	ACAAGAGCAGAAGGCGAATGGC	GTGCAGCAGGGACAGTGTAAGC
*TSLP*	GGTGCCCAGGCTATTCGGAAAC	TGAAGCGACGCCACAATCCTTG
*OPG*	GCCCCTTGCCCTGACCACTAC	TGCGATTGCACTCCTGCTTGAC
*OX40*	GCTCGGACGCAATCTGTGAGG	AGTGGGCTGGACAGTGATGGG
*TNF*	CGTGGAGCTGGCCGAGGAG	GCAGGCAGAAGAGCGTGGTG
*CLCF1*	GACTTGGAGGTGTGGCGAAGC	CAGTGGCAGCCTGACGGTTG
*CCL26*	CCACCTTGGAACTGCCACACG	TAGCTTCGCACCCAGGTCCAG
*CCR4*	GGCTACATGGTCAGTGGCTGTG	TCCAGGGAGCTGAGAACCTTCC

*GAPDH* glyceraldehyde-3-phosphate dehydrogenase, *CREB3L3* cAMP responsive element binding protein 3 like 3, *NPR1* natriuretic peptide receptor 1, *TRPC6* transient receptor potential cation channel subfamily C member 6, *ATP1A4* ATPase Na^+^/K^+^ transporting subunit alpha 4, *ADRA1D* adrenoceptor alpha 1D, *CACNA1D* calcium voltage-gated channel subunit alpha 1D, *DAN MINOS1-NBL1*, MINOS1-NBL1 readthrough, *PITX2* paired like homeodomain 2, *ALK7 ACVR1C*, activin A receptor type 1C, *CCL4* C–C motif chemokine ligand 4, *VEGI TNFSF15*, TNF superfamily member 15, *IL21R* interleukin 21 receptor, *TGFβ2* transforming growth factor beta 2, *TSLP* thymic stromal lymphopoietin, *OPG TNFRSF11B*, TNF receptor superfamily member 11b, *OX40 TNFRSF4*, TNF receptor superfamily member 4, *TNF* tumor necrosis factor, *CLCF1* cardiotrophin like cytokine factor 1, *CCL26* C–C motif chemokine ligand 26, *CCR4* C–C motif chemokine receptor 4

### Western blot analysis

According to the sequencing data and functional analysis, we analyzed the proteins associated with the transforming growth factor beta (TGF-β) signaling pathway in granulosa cells using the Western blot. Briefly, the whole protein samples were extracted, and their concentrations measured using the BCA Protein Assay Kit (GenStar, Beijing, China). Then, equal amounts of proteins (20 μg per lane) were loaded and separated on a 12% sodium dodecyl sulfate (SDS) polyacrylamide gel. Primary antibodies were used after proteins transferred to the 0.45-μm Immobilon-P PVDF Membrane, and GAPDH used as loading control. Signals were obtained in the linear range of detection and quantified with the Bio-Rad ChemiDoc Imaging System. The grey values of target bands were analyzed by ImageJ software and normalized to the reference band. The primary antibodies used were TGF-β2 (19,999–1-AP; 1:2000 diluted; Proteintech), TGF-βR2 (66,636–1-lg; 1:2000 diluted; Proteintech), Smad2 (12,570–1-AP; 1:2000 diluted; Proteintech), Smad3 (25,494–1-AP; 1:2000 diluted; Proteintech).

### Hormone assay of follicular fluid and correlation analysis

The FF was thawed to RT and fully mixed before analysis. The FF anti-müllerian hormone (AMH), FSH, LH, E_2_, progesterone (P), testosterone (T) and prolactin (PRL) were measured by electrochemiluminescence immunoassay (ECLIA) following the instructions (Roche Diagnostics GmbH, Mannheim, Germany). The TGF-β2, bone morphogenetic protein-15 (BMP-15) and growth differentiation factor-9 (GDF-9) concentrations were detected using enzyme-linked immunosorbent assay (ELISA). And the correlation analysis between the cytokine concentrations and the laboratory outcomes was performed.

### Statistical analysis

All clinical parameters and intrafollicular hormonal concentration values were expressed as means ± standard deviation (SD). Differences in the laboratory outcomes and the intrafollicular hormone concentrations between the two groups were compared via Student’s t-test. The statistical analyses of the Western blot and RT-qPCR results were performed using Student’s t-test. Spearman’s correlation coefficient was calculated to detect the correlation between the TGF-β2 level in the FF and the rate of the good-quality embryo. Analyses were performed using SPSS (version 23, Chicago, USA), and significance defined as a *P* value < 0.05.

## Results

### Clinical characteristics and outcomes of patients

Table 2 shows the clinical characteristics of the recruited 48 POR patients, who were categorized into two groups, i.e., the mild group (*n* = 27) and the COS group (*n* = 21), with no statistical differences in age, BMI, serum AMH and basal hormonal levels. Compared with the COS group, both the Gn stimulation duration and the total Gn dosage were significantly shorter and lower in the mild group, and also the serum E_2_ level on trigger day statistically lower. Although a higher number of retrieved oocytes were obtained in the COS group, no statistical differences observed regarding the numbers of the metaphase II oocyte, 2 pronuclei (2PN), transferrable embryo and good-quality embryo. Noteworthily, significantly higher rates of the metaphase II oocyte and the good-quality embryo were observed in the mild group (Table 2).

**Table 2:** Clinical characteristics and outcomes of the two groups

	Mild group	COS group	*P* value
Age (years)	39.19 ± 3.25	38.05 ± 3.72	0.264
AMH (ng/ml)	0.61 ± 0.30	0.77 ± 0.22	0.053
BMI (kg/m^2^)	23.08 ± 2.66	21.70 ± 1.92	0.051
Basal FSH (U/L)	8.68 ± 2.90	9.67 ± 5.27	0.415
Basal E_2_ (pg/ml)	54.23 ± 21.28	48.38 ± 18.75	0.326
Basal LH (U/L)	4.18 ± 1.42	4.32 ± 2.06	0.783
Duration of stimulation (days)	5.93 ± 1.64	10.67 ± 1.49	< 0.001^a^
Total gonadotropin/cycle (IU)	867.59 ± 241.37	3152.38 ± 408.19	< 0.001^a^
Serum E_2_ level on trigger day (pg/ml)	522.33 ± 325.11	1205.13 ± 504.42	< 0.001^a^
Serum LH level on trigger day (U/L)	5.94 ± 5.43	2.91 ± 1.52	0.017^a^
Serum P level on trigger day (ng/ml)	0.47 ± 0.35	0.79 ± 0.41	0.006^a^
No. of oocytes retrieved	3.48 ± 1.40	5.29 ± 3.50	0.018^a^
No. of oocytes in metaphase II	2.70 ± 1.43	3.29 ± 2.70	0.342
Rate of oocytes in metaphase II (%)	77.66	62.16	0.017 ^a^
No. of 2PN oocytes	2.22 ± 1.28	3.14 ± 2.59	0.114
No. of transferrable embryos	2.00 ± 1.24	2.48 ± 2.02	0.319
No. of good-quality embryos	1.83 ± 1.24	1.76 ± 1.68	0.881
Rate of good-quality embryos (%)	75.86	57.69	0.043 ^a^

Values expressed as means ± SD or percentage. *AMH* anti-müllerian hormone, *BMI* body mass index, *FSH* follicle stimulating hormone, *E*_*2*_ estradiol, *LH* luteinizing hormone, *P* progesterone; *2PN* 2 pronuclei

^a^Results are significantly different between the two groups

### Transcriptional profiles between samples from the two groups

**Fig. 1:**
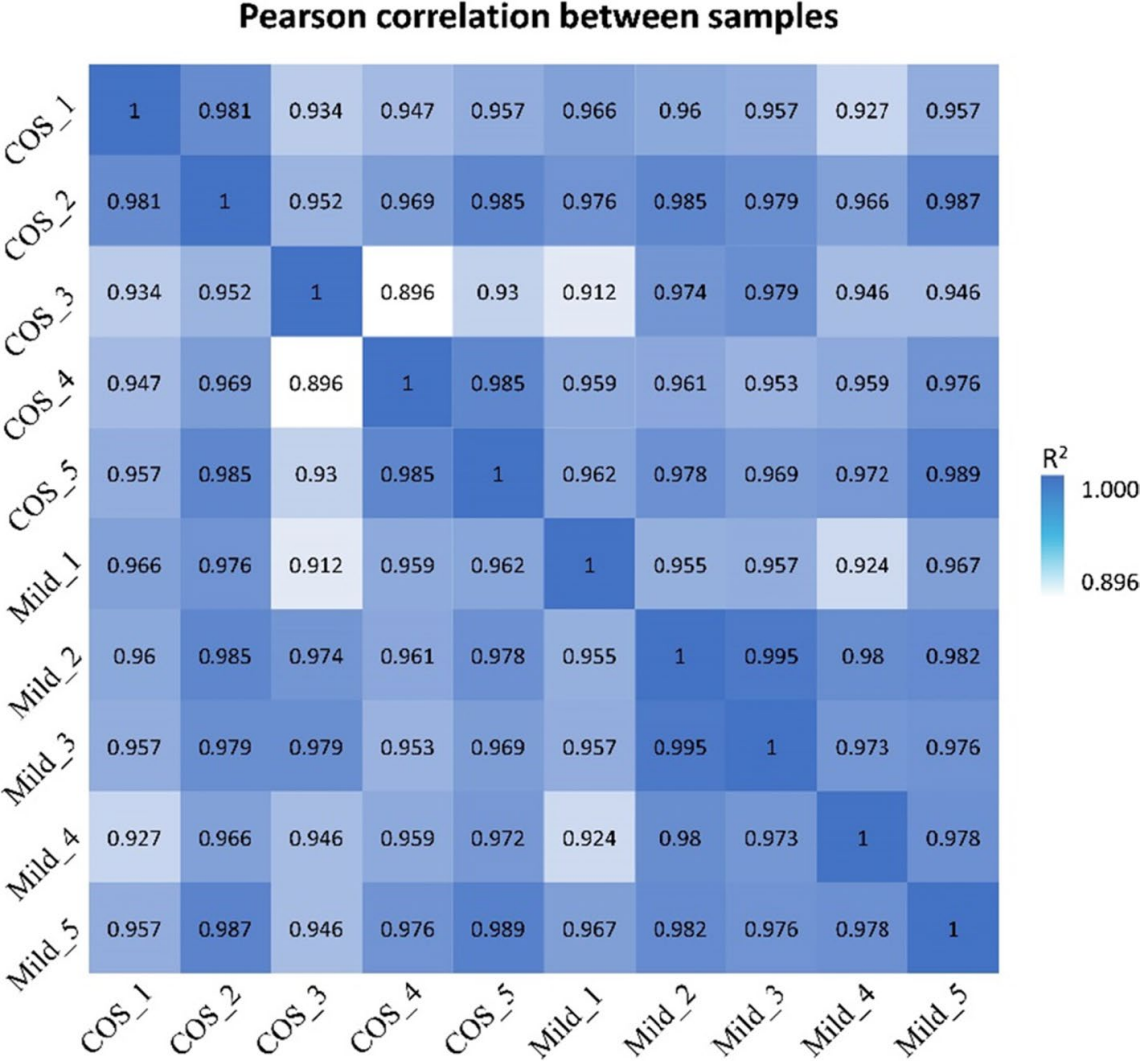
Pearson correlation analysis between samples. The color key from blue to white indicates the correlation from high to low

We obtained 55–67 million 150 bp reads for each sample, and 92 Gb of raw data in total were obtained for all the samples. Clean data, which were used for subsequent analyses, were ~ 8 Gb per sample (Supplementary data Table S1). We calculated the Pearson correlation coefficient of every two samples by using the gene expression. As shown in Fig. 1, the expression patterns were generally homogeneous between every two samples, all *R*^*2*^ > 0.9 except for COS_3 and COS_4 (*R*^*2*^ = 0.896).

### Analyses of DEGs and function enrichment

**Fig. 2:**
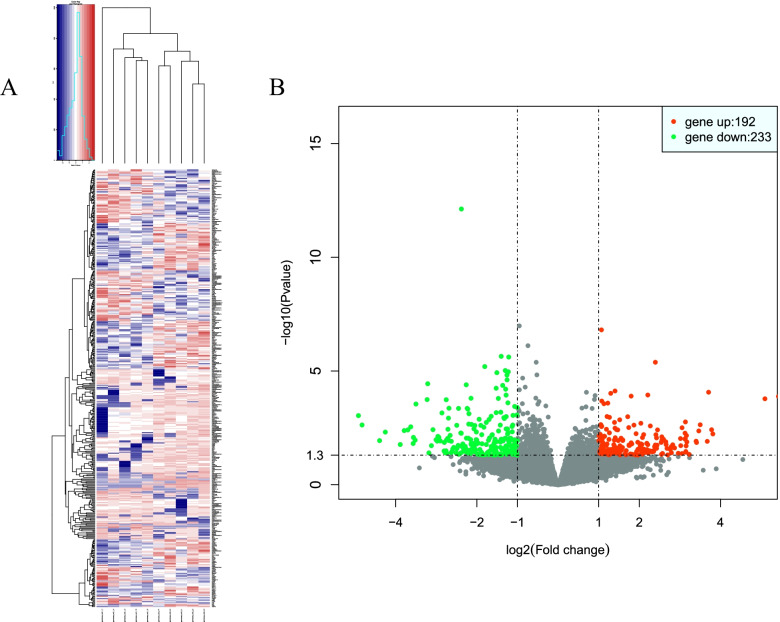
Cluster and filter analyses of DEGs. **A** Heatmap of the DEGs between the two groups. The color key from blue to red indicates the relative gene expression level from low to high. **B** Volcano map showing the DEGs. The x-axis shows the foldchange in gene expression, and the y-axis shows significant statistical differences. Red dots represent up-regulated genes, and green dots represent down-regulated genes

The clustering analysis was used to determine the DEGs (Fig. 2A) between the two groups, and totally 425 genes were found to be differentially expressed, with 192 being up-regulated and 233 down-regulated in the mild group (*P* < 0.05, absolute value of log_2_(Foldchange) > 1) (Fig. 2B).

**Fig. 3:**
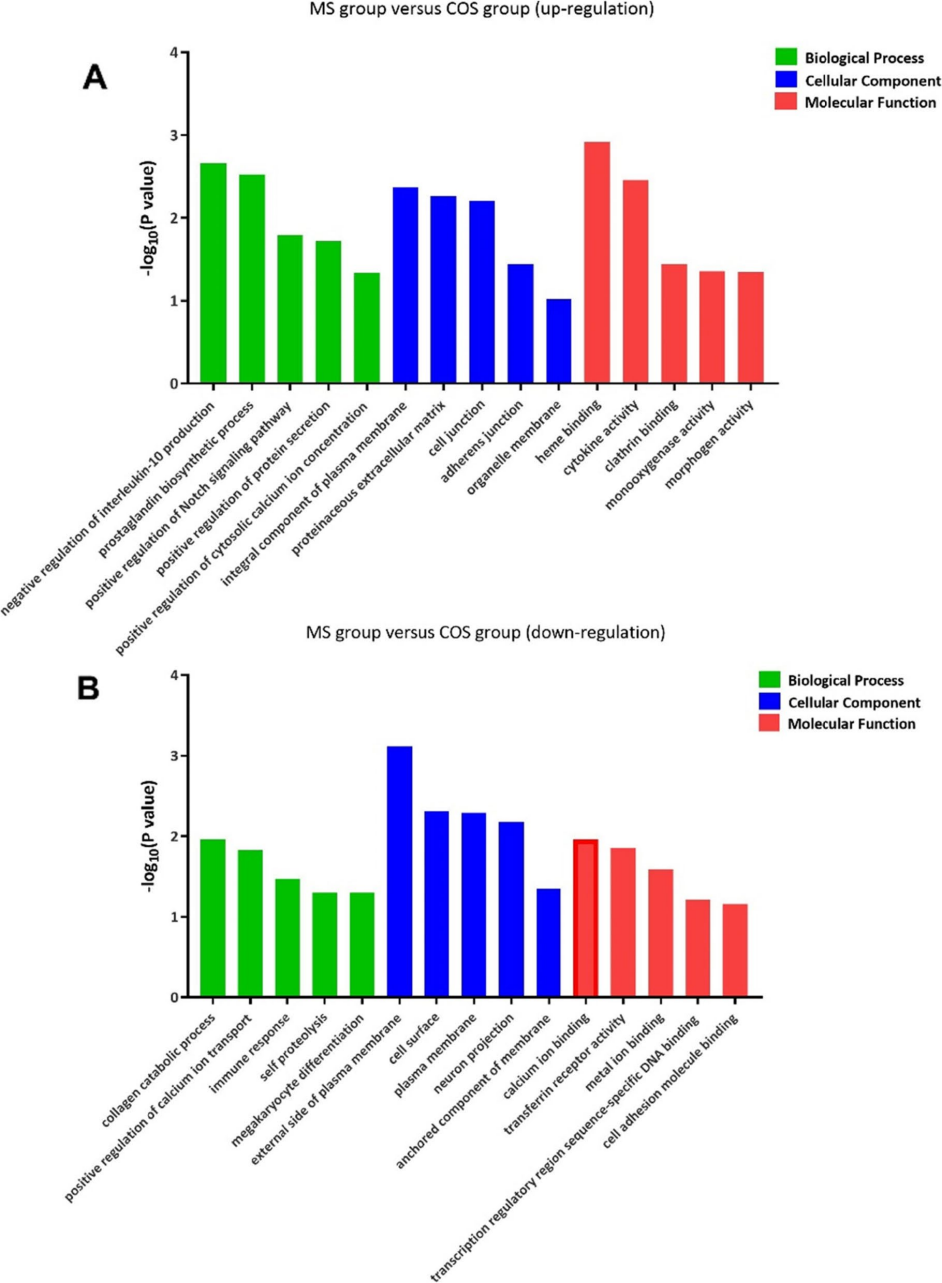
GO enrichment analysis of DEGs between the two groups. The x-axis shows the five representative enrichment terms in three categories, including biological process in green bars, cellular component in blue bars and molecular function in red bars. The y-axis represents -log_10_(*P* value). Compared with COS group, **A** genes up-regulated in mild group, and **B** genes down-regulated in mild group

The GO enrichment analysis was performed to explore the biological functions of the 425 DEGs, and three categories of biological functions were identified, including biological process, cellular component, and molecular function. Five representative GO enrichment results in each category are presented in Fig. 3. Fig. 3A shows the up-regulated DEGs in the mild group, which are related to cytokine activity and regulation activity, and Fig. 3B shows the down-regulated DEGs in the mild group, which are involved in the function of immune response.

The 425 DEGs were also assigned to the KEGG enrichment analysis, and Table 3 shows the top ten pathways (*P* < 0.05), among which are some associated with the oocyte development, including the cytokine-cytokine receptor interaction, TGF-β signaling pathway, cGMP-PKG signaling pathway, and metabolic pathway.

**Table 3:** Top ten KEGG pathways of DEGs between the two groups

Pathway Id	Name of KEGG pathway	No. of genes	Genes	*P*-Value
hsa04060	Cytokine-cytokine receptor interaction	13	*CCR4; CCL26; CLCF1; TNF; TNFRSF4; TNFRSF11B; TSLP; IL24; TGF-β2; IL21R; TNFSF15; MPL; CCL4*	2.46E-07
hsa05410	Hypertrophic cardiomyopathy	5	*TNF; ITGA2B; CACNA1D; DES; TGF-β2*	5.62E-04
hsa04350	TGF-β signaling pathway	5	*TNF; ACVR1C; PITX2; MINOS1-NBL1; TGF-β2*	5.92E-04
hsa04640	Hematopoietic cell lineage	5	*TNF; TFRC; GP9; CD1B; ITGA2B*	7.23E-04
hsa05414	Dilated cardiomyopathy	5	*TNF; ITGA2B; CACNA1D; DES; TGF-β2*	7.96E-04
hsa04022	cGMP-PKG signaling pathway	6	*CACNA1D; ADRA1D; ATP1A4; TRPC6; NPR1; CREB3L3*	2.12E-03
hsa00512	Mucin type O-Glycan biosynthesis	3	*WBSCR17; GALNT15; GALNT8*	2.15E-03
hsa04976	Bile secretion	4	*CA2; CYP3A4; ABCB1; ATP1A4*	2.56E-03
hsa05412	Arrhythmogenic right ventricular cardiomyopathy	4	*CTNNA2; ITGA2B; CACNA1D; DES*	2.95E-03
hsa01100	Metabolic pathways	19	*WBSCR17; RIMKLA; INPP5J; CYP3A7; CEL; PTGS2; PTGIS; IDO1; NDST4; GALNT15; NMNAT2; CYP3A4; ST6GALNAC5; GALNT8; PLA2G3; ARG1; POLR2J2; TKTL1; AMDHD1*	3.74E-03

### RT-qPCR validation

**Fig. 4:**
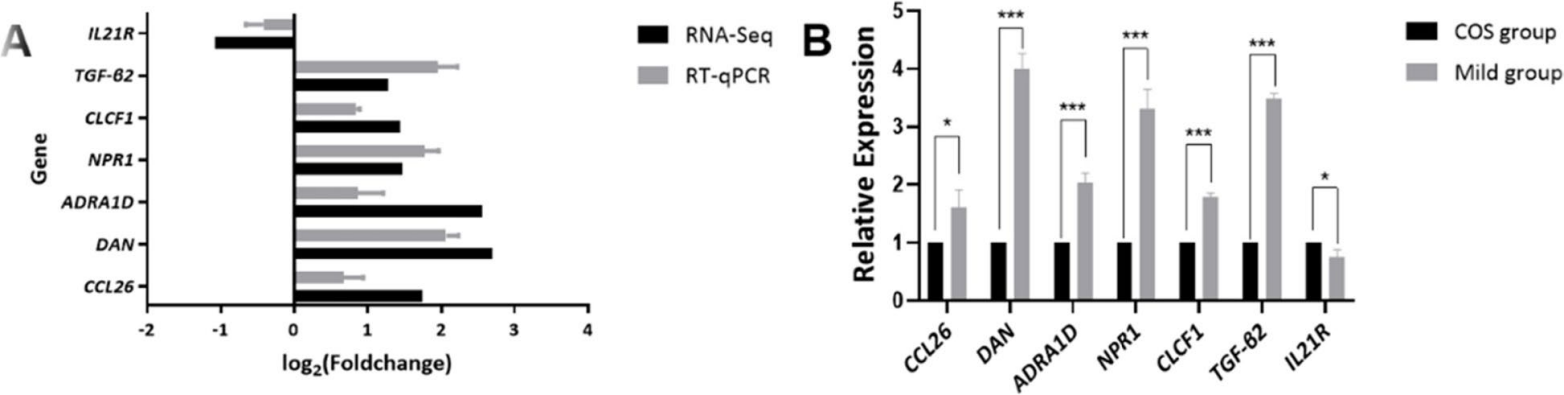
Validation of the RNA sequencing results using RT-qPCR. **A** Bars extending to the right or left of zero coordinate represent up or down regulated genes, respectively. Black bars represent expression of transcript in RNA sequencing while grey bars represent expression of the same transcript presented by RT-qPCR. **B** Comparison of expression of 7 selected genes between the two groups. Asterisk (*) above the bar represents statistical difference in RT-qPCR experiments. *P < 0.05; ***P < 0.001

Based on the sequencing data and functional analyses, 20 genes were selected for validation with RT-qPCR not only according to the most significant fold change variations but also in terms of the role they play in the pathway associated with the oocyte formation and development (Table 1). Of the 20 genes, 6 genes (*TGF-β2, CLCF1, NPR1, ADRA1D, DAN, CCL26*) exhibited by RNA sequencing in the mild group were confirmed in upregulation and 1 gene (*IL21R*) confirmed downregulation (Fig. 4).

### Protein expressions in granulosa cells

**Fig. 5:**
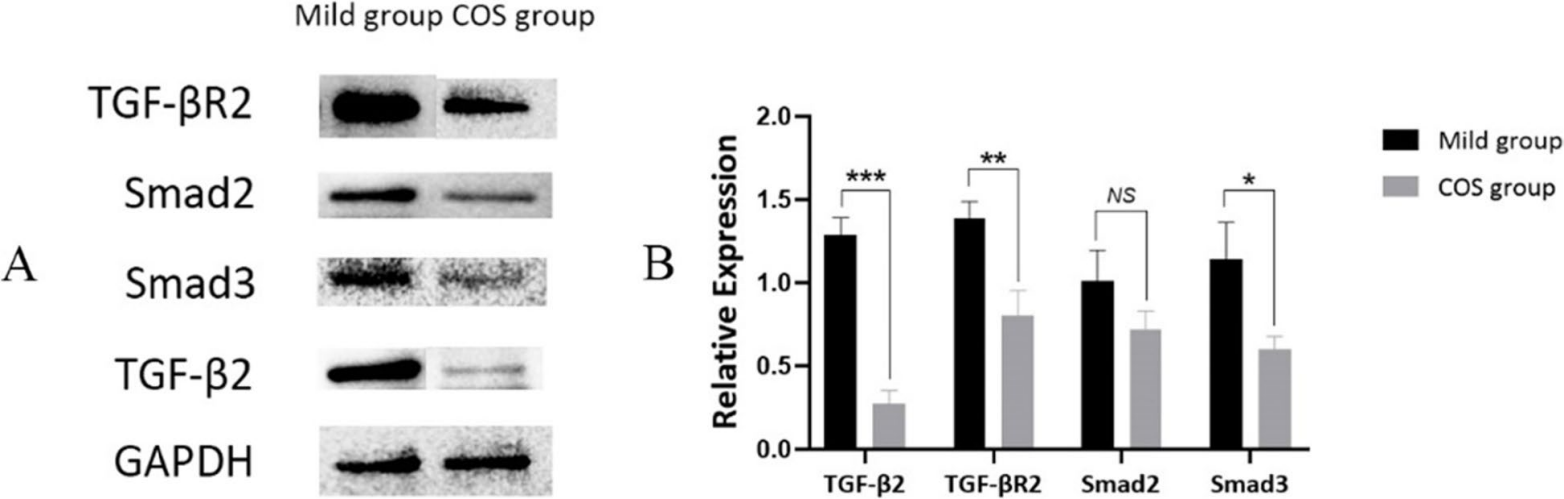
Western blot analysis of TGF-β signaling pathway specific proteins TGF-β2, TGF-βR2, Smad2 and Smad3 in the mild and COS groups. GAPDH used as a control. **A** Western blot results. **B** Quantity analysis based on the Western blot results. Mild group (*n* = 10), and COS group (*n* = 8). *P < 0.05; **P < 0.01; ***P < 0.001; *NS* No significant

To determine the different protein expression levels of the TGF-β signaling pathway in granulosa cells between the two groups, such protein products as TGF-β2, TGF-βR2, Smad2 and Smad3 were detected. The expression of TGF-β2, TGF-βR2 and Smad3 in the mild group were significantly upregulated compared with the COS group (Fig. 5).

### Follicular fluid analysis and correlation analysis

**Fig. 6:**
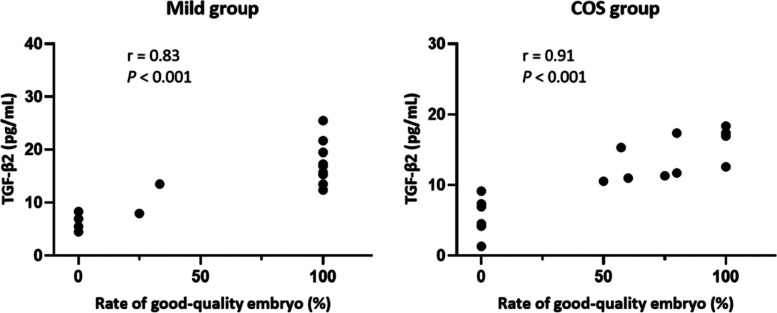
Spearman correlation analysis between the TGF-β2 level in the FF and the rate of the good-quality embryo. The scatter diagrams of the two groups show the monotonic relationship between the TGF-β2 level and the rate of the good-quality embryo, r is correlation coefficient and *P* < 0.05 indicates that the correlation is statistically significant

Different hormone and cytokine concentrations in the follicular fluid (FF) of the two groups are listed in Table 4. Compared with the COS group, the FSH, PRL and P levels in the FF were significantly lower in the mild group while the LH and T levels significantly higher. As for the cytokine concentrations, a significantly higher TGF-β2 concentration and a significantly lower GDF-9 level were detected in the mild group while the BMP-15 levels were comparable between the two groups. Correlation analysis revealed that there was a positive correlation between the TGF-β2 concentration and the rate of the good-quality embryo (Fig. 6).

**Table 4:** Hormone and cytokine concentrations in FF between the two groups

	Mild group	COS group	*P* value
AMH (ng/ml)	1.05 ± 0.51	1.54 ± 1.02	0.095
FSH (IU/L)	4.76 ± 1.55	9.80 ± 3.27	< 0.001^a^
E_2_ (ng/ml)	394.97 ± 262.84	325.67 ± 169.20	0.390
LH (IU/L)	6.80 ± 4.65	1.02 ± 0.99	< 0.001^a^
PRL (ng/ml)	33.34 ± 18.79	58.49 ± 29.64	0.007^a^
T (ng/ml)	45.59 ± 21.03	5.47 ± 2.19	< 0.001^a^
P (μg/ml)	37.64 ± 21.95	79.58 ± 21.41	< 0.001^a^
TGF-β2 (pg/ml)	21.21 ± 10.51	11.89 ± 8.21	0.014 ^a^
GDF-9 (pg/ml)	61.22 ± 17.62	194.90 ± 56.47	< 0.001^a^
BMP-15 (pg/ml)	42.60 ± 7.01	41.67 ± 11.18	0.777

Values expressed as means ± SD. *AMH* anti-müllerian hormone, *FSH* follicle stimulating hormone, *E2* estradiol, *LH* luteinizing hormone, *PRL* prolactin, *T* testosterone, *P* progesterone, *TGF-β2* transforming growth factor-β2, *GDF-9* growth differentiation factor-9, *BMP-15* bone morphogenetic protein-15

^a^Results are significantly different between the two groups

## Discussion

**Fig. 7:**
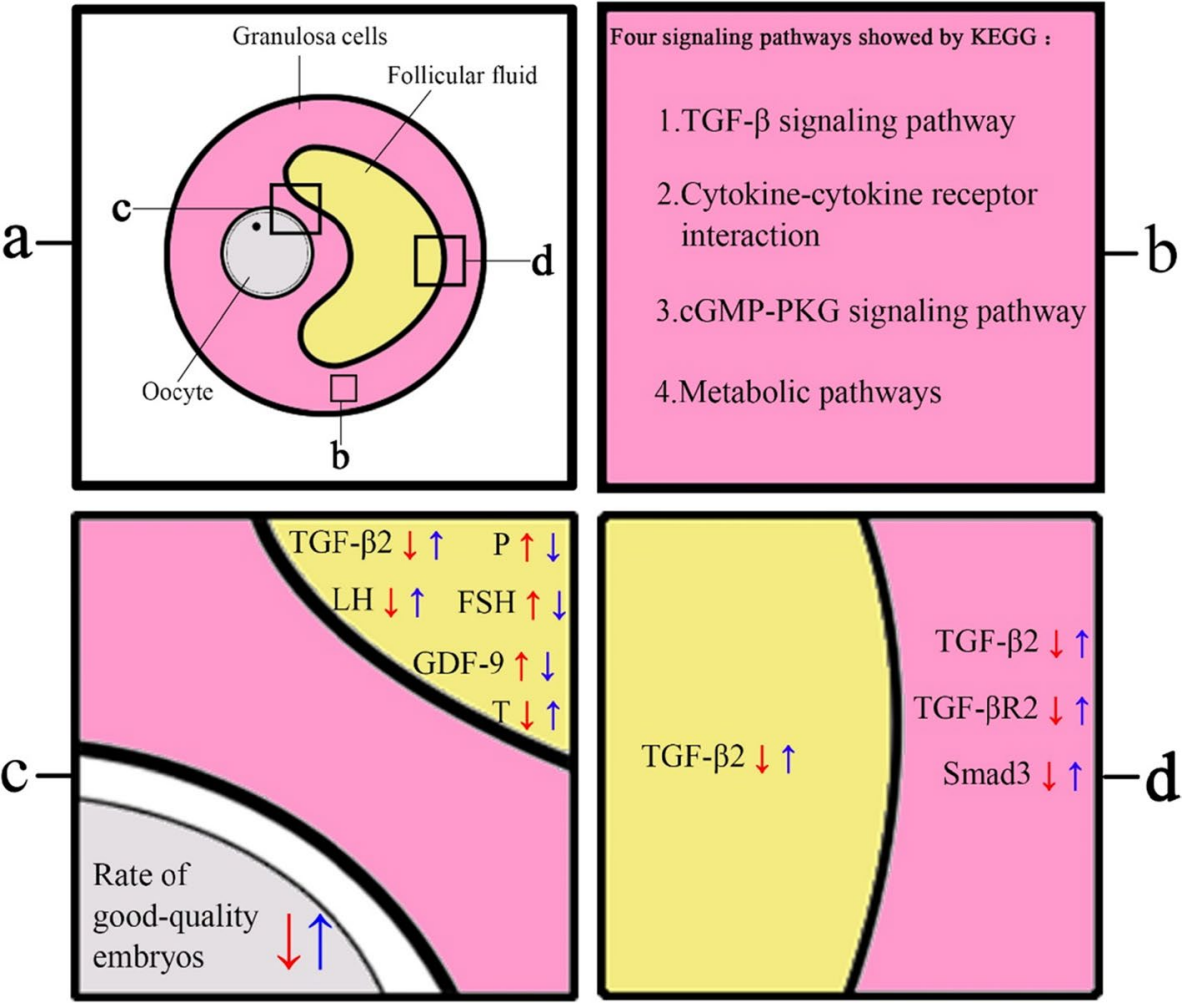
Schematic diagram of the results in the background of cross-talkings within the follicle. The red and blue arrows represent the COS group and the mild group, respectively. The up and down arrows represent up-regulation and down-regulation, respectively. **a** Histologic and functional architecture of a mature follicle. Panels **b**, **c**, and **d** summarize the main findings of this study. **b** The KEGG analysis of DEGs between the two groups. After sequencing and analyzing the DEGs of granulosa cells from the two groups, the KEGG analysis showed four signaling pathways associated with the oocyte development. **c** Comparative analyses of the rate of good-quality embryos as well as the hormones and cytokines in the FF. Significantly higher rates of good-quality embryos were observed in the mild group. Compared with the COS group, the FSH and P levels in the FF were significantly lower in the mild group while the LH and T levels significantly higher. As for the cytokine concentrations, a significantly higher TGF-β2 concentration and a significantly lower GDF-9 level were detected in the mild group. **d** Comparative analyses of TGF-β2, TGF-βR2 and Smad3 in granulosa cells as well as TGF-β2 in the FF. The expressions of TGF-β2, TGF-βR2 and Smad3 in the mild group were significantly upregulated compared with the COS group

The follicle, underlying the follicular development, oocyte maturation, cumulus expansion and ovulation, is a histological and functional architecture supporting a series of complicated, subtle and continuous cross-talkings among the oocyte, the somatic follicular cells (particularly the granulosa cell) and the FF micro-environment [24] (Fig. 7). Granulosa cells have close relationship with the oocyte, affecting the oocyte development and contributing to the FF micro-environment through a series of dynamic regulations in levels of the transcription, protein and metabolism [25]. The FF components may be directly altered by the hormonal, paracrine and autocrine signaling pathways [26], which, further, has been suggested to influence the oocyte quality, early embryo development and subsequent pregnancy [27, 28]. Nowadays, as an indispensable medicine for ovarian stimulation in the ART practice, exogenous Gn is used to promote follicular growth via its influence on the cross-talking among the oocyte, granulosa cell and FF. Gn dose is the main difference in our two protocols, but the effect of this difference on the oocyte development and the mechanism underlying which it affects the oocyte development remain unclear although Lu et al. showed that Gn stimulation may potentially induce meiotic errors of human oocytes through analyzing the transcriptome of the granulosa cell after natural and Gn stimulation cycles [29].

The results of this study showed that the DEGs between the two protocols were mainly involved in the TGF-β signaling pathway, cytokine-cytokine receptor interactions, metabolic pathways and cGMP-PKG signaling pathway, all of which are involved in the developmental processes of the follicle and oocyte. The RNA sequencing result, qRT-PCR result and Western blot analysis revealed the up-regulation of the genes and proteins related to the TGF-β signaling pathway in the mild group, and a higher rate of the good-quality embryo also observed in the mild group. The correlation analysis further confirmed a positive correlation between the TGF-β2 level in the FF and the rate of the good-quality embryo. These results imply that the TGF-β signaling pathway may be closely associated with the embryo quality, which is consistent to literatures reporting that the TGF-β signaling pathway was directly related to early embryonic development and blastocyst formation in bovine, and affected the oocyte by mediating the proliferation of granulosa cells [30–33].

As an important link within the cross-talking among the oocyte, granulosa cell and FF, the TGF-β signaling pathway is under complicated regulations of the FF components, which has not been completely addressed. In this study, lower intrafollicular FSH was found in the mild group due to the use of fewer doses of Gn. Although the clinical significance of lower FSH remains uncertain, previous study pointed out that high-dose Gn stimulation could impair embryo development potential [34], which is supported by our study, showing a higher rate of the good-quality embryo in the mild group. And, in our experimental system, through detecting the granulosa cell and the FF from the same individual sample, observed in the mild group were a significant up-regulation of the TGF-β signaling pathway-related genes and proteins in the cells, and a decrease of FSH along with the increase of LH and T in the FF. However, Gueripel et al. showed that the increase of FSH and LH could up-regulate the TGF-β signaling pathway in immature mice after Gn stimulation [35]. This inconsistence can be explained in the following aspects. First, a major difference between us lies in that we used letrozole in the mild group. Letrozole, an aromatase inhibitor blocking the transformation of androstenedione into estrogen, will cause different T level in the FF, and T could affect the expression of TGF-β signaling [36]. Second, the granulosa cell and the FF in our study were sampled during ovulation while their samples were from the follicular phase, and the different time windows will present different observation facts because the sexual hormones relevant to the follicular development appear and effect in terms of a periodical and dynamic regulating axis. Thirdly, the different experimenting systems, such as measurement methods and experiment species, may also contribute to the inconsistence.

We also measured the cytokines relevant to the oocyte development in the FF, i.e., TGF-β2, BMP-15 and GDF-9. Comparable BMP-15 levels were found in the two groups while a significantly lower GDF-9 and a significantly higher TGF-β2 in the FF were found in the mild group, whose oocyte maturity rate and good-quality embryo rate were both higher. This observation is inconsistent to the previous reports [37, 38] showing that higher GDF-9 and BMP-15 levels in the FF were significantly correlated with a higher oocyte maturation and a better embryo quality, which can be explained as follows: 1) The increase of GDF-9 is positively correlated with the increase of FSH [39], and the higher level of FSH in the COS group accounted for the higher level of GDF-9; 2) Only if the heterodimer between GDF-9 and BMP-15 has been structured was there any biological activity, such as activating downstream molecules, promoting the proliferation of granulosa cells and regulating the growth and development of oocytes [40]. Therefore, the measurement of GDF-9 or BMP-15 levels alone does not accurately reflect the quality of embryos.

Here are three limitations in this study. First, only the mural granulosa cells were sampled for the transcriptome data analysis while the cumulus granulosa cells were spared because of the difficulty in obtaining enough cells for sequencing. Second, morphologic assessment rather than preimplantation genetic testing (PGT) was applied to rating the quality of embryos because PGT can only be used for such specific patients as with genetic diseases, recurrent pregnancy loss, repeated IVF failure, etc., according to the regulations of Chinese health administration. A slight concern is that GnRH agonist and GnRH antagonist were used in our study. But, according to literatures [41, 42], different GnRH analogs make small difference in the granulosa cell at the transcriptome level, meaning that they could not undermine our conclusion. In the future, we would like to validate biological functions of the DEGs and reveal the underlying molecular mechanisms.

## Conclusions

Collectively, based on the transcriptome sequencing technique, after comparing the gene and protein expression in the granulosa cell and the alteration in the FF components from the samples of POR patients with the mild ovarian stimulation or the conventional COS, we found that the TGF-β2 signaling pathway was correlated with the good quality of oocytes, and played a crucial role in the cross-talking among the three of the granulosa cell, FF and oocyte, which implicates that the mild ovarian stimulation protocol is more beneficial to POR patients.

## Supplementary Information

**Additional file 1. **

**Additional file 2. **

## Data Availability

Sequence data from this article have been deposited with the GEO under accession number GSE191322.

## Abbreviations

IVF: In in Vitro Fertilization
COS: Controlled Ovarian Stimulation
POR: Poor Ovarian Response
Gn: Gonadotropin
LH: Luteinizing Hormone
GnRH-a: Gonadotropin-Releasing Hormone agonist
FF: Follicular Fluid
FSH: Follicle Stimulation Hormone
hCG: Human Chorionic Gonadotropin
E_2_: Estradiol
BMI: Body Mass Index
PBS: Phosphate Buffered Saline
RT: Room Temperature
DEGs: Differentially Expressed Genes
*P*_*adj*_: Adjusted *P*-values
GO: Gene Ontology
KEGG: Kyoto Encyclopedia of Genes and Genomes
RT-qPCR: Real-Time Quantitative PCR
GAPDH: Glyceraldehyde-3-Phosphate Dehydrogenase
TGF-β: Transforming Growth Factor Beta
SDS: Sodium Dodecyl Sulfate
AMH: Anti-Müllerian Hormone
P: Progesterone
T: Testosterone
PRL: Prolactin
ECLIA: Electrochemiluminescence Immunoassay
BMP-15: Bone Morphogenetic Protein-15
GDF-9: Growth Differentiation Factor-9
ELISA: Enzyme-Linked Immunosorbent Assay
SD: Standard Deviation
2PN: 2 Pronuclei
PGT: Preimplantation Genetic Testing
