# Supplementary material for: Comparative analyses in transcriptome of human granulosa cells and follicular fluid micro-environment between poor ovarian responders with conventional controlled ovarian or mild ovarian stimulations
Source: Reprod Biol Endocrinol. 2022 Mar 21;20:54. doi: 10.1186/s12958-022-00926-1 (PMC8935846; doi:10.1186/s12958-022-00926-1)
Supplement: Supplementary file 1 — Additional file 1. [file 12958_2022_926_MOESM1_ESM.docx]

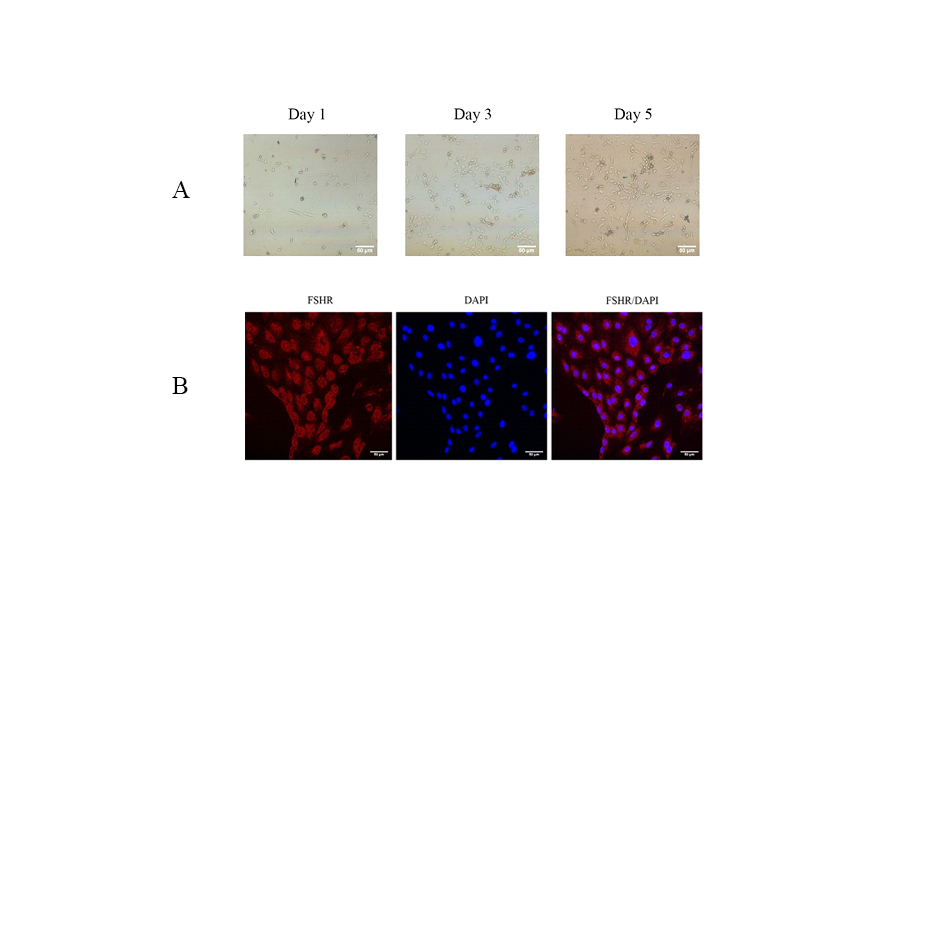


**Figure S1** Granulosa cells identification. **A** Granulosa cells culture (Scale: 50 μm). **B** Granulosa cells FSHR immunofluorescence staining (red: FSHR staining; blue: DAPI staining). The red fluorescence expression in cytoplasm indicated that the cells were granulosa cells specifically expressing FSHR (Scale: 50 μm).
