# Supplementary material for: Comparative analyses in transcriptome of human granulosa cells and follicular fluid micro-environment between poor ovarian responders with conventional controlled ovarian or mild ovarian stimulations
Source: Reprod Biol Endocrinol. 2022 Mar 21;20:54. doi: 10.1186/s12958-022-00926-1 (PMC8935846; doi:10.1186/s12958-022-00926-1)
Supplement: Supplementary file 2 — Additional file 2. [file 12958_2022_926_MOESM2_ESM.docx]

Table S1. Summary of sequence assembly after Illumina sequencing

| **Samples** | **Raw reads** | **Clean reads** | **Clean bases** | **Error rate (%)** | **Q20 (%)** | **Q30 (%)** | **GC content (%)** |
| --- | --- | --- | --- | --- | --- | --- | --- |
| Mild_1_r1 | 28,598,380 | 26,863,244 | 3,937,543,836 | 0.20 | 97.50 | 93.62 | 49.50 |
| Mild _1_r2 | 28,598,380 | 26,863,244 | 3,845,606,764 | 0.45 | 94.40 | 87.84 | 49.65 |
| Mild _2_r1 | 27,799,383 | 25,944,873 | 3,776,516,423 | 0.23 | 96.98 | 92.88 | 48.07 |
| Mild _2_r2 | 27,799,383 | 25,944,873 | 3,686,308,975 | 0.48 | 93.83 | 87.07 | 48.17 |
| Mild _3_r1 | 29,815,134 | 27,836,399 | 4,059,469,986 | 0.22 | 97.09 | 93.03 | 48.12 |
| Mild _3_r2 | 29,815,134 | 27,836,399 | 3,963,275,422 | 0.48 | 93.93 | 87.13 | 48.26 |
| Mild _4_r1 | 31,526,907 | 29,445,289 | 4,309,613,711 | 0.21 | 97.30 | 93.34 | 48.78 |
| Mild _4_r2 | 31,526,907 | 29,445,289 | 4,207,093,234 | 0.47 | 94.11 | 87.42 | 48.93 |
| Mild _5_r1 | 28,690,154 | 26,862,171 | 3,935,188,029 | 0.20 | 97.38 | 93.46 | 49.71 |
| Mild _5_r2 | 28,690,154 | 26,862,171 | 3,840,822,649 | 0.46 | 94.22 | 87.58 | 49.84 |
| COS_1_r1 | 27,722,533 | 25,994,393 | 3,814,351,960 | 0.20 | 97.48 | 93.63 | 49.72 |
| COS_1_r2 | 27,722,533 | 25,994,393 | 3,723,292,935 | 0.45 | 94.31 | 87.74 | 49.85 |
| COS_2_r1 | 33,152,746 | 30,907,087 | 4,539,175,404 | 0.20 | 97.46 | 93.53 | 49.94 |
| COS_2_r2 | 33,152,746 | 30,907,087 | 4,421,616,429 | 0.48 | 93.94 | 87.00 | 50.10 |
| COS_3_r1 | 33,273,902 | 31,137,815 | 4,555,544,993 | 0.20 | 97.45 | 93.56 | 49.27 |
| COS_3_r2 | 33,273,902 | 31,137,815 | 4,445,821,802 | 0.46 | 94.21 | 87.52 | 49.42 |
| COS_4_r1 | 33,334,398 | 31,280,492 | 4,573,371,104 | 0.20 | 97.38 | 93.50 | 48.50 |
| COS_4_r2 | 33,334,398 | 31,280,492 | 4,465,744,148 | 0.45 | 94.31 | 87.81 | 48.64 |
| COS_5_r1 | 31,336,660 | 29,313,799 | 4,292,810,066 | 0.20 | 97.38 | 93.45 | 49.39 |
| COS_5_r2 | 31,336,660 | 29,313,799 | 4,189,562,499 | 0.46 | 94.18 | 87.50 | 49.55 |

Notes: Q20, percentage of bases with a Phred value of at least 20; Q30, percentage of bases with a Phred value of at least 30.
